# Source-controlled bacterial peritonitis improves survival but leaves persistent lung inflammation and airway IgA loss

**DOI:** 10.1186/s40635-026-00931-3

**Published:** 2026-06-22

**Authors:** Azusa Kato, Masafumi Saito, Hiromi Miyazaki, Bradley M Kearney, Masahiro Nakashima, Hiroyuki Nakashima, Kaoru Koyama, Manabu Kinoshita

**Affiliations:** 1 Department of Anesthesiology, Saitama Medical Center, Saitama Medical University, Kawagoe, Japan; 2 Department of Immunology and Microbiology, National Defense Medical College, Tokorozawa, Japan; 3 Division of Biomedical Engineering, National Defense Medical College Research Institute, Tokorozawa, Japan; 4 Department of Veterinary Pathology, College of Veterinary Medicine, Iowa State University, Ames, IA USA

**Keywords:** Sepsis, Source control, Post-acute phase, Lung inflammation, Mucosal immunity, IgA, Airway epithelial injury

## Abstract

**Background:**

Cecal ligation and puncture (CLP) is widely used to develop polymicrobial sepsis model in rodents, yet conventional CLP is not advantageous for evaluating long-term outcomes because most animals succumb without clinically aligned treatment. We therefore implemented source control (SC) combined with antibiotic therapy after CLP to enable post-acute observation and characterize pulmonary immune alterations after abdominal sepsis.

**Methods:**

Twenty-eight-week-old male C57BL/6J mice underwent CLP. After 6 h, the necrotic cecum was resected, followed by peritoneal lavage, and antibiotics were administered for three days (SC group). Sham mice underwent matched laparotomies without CLP, and age-matched naïve mice that did not undergo surgery or treatment served as controls. Survival, systemic inflammation, bacterial burden, and organ injury were assessed up to day 14 after CLP. We also evaluated pulmonary inflammation, lung immune cell composition, immunoglobulin profiles in plasma, lung homogenates, and bronchoalveolar lavage fluid (BALF), as well as airway epithelial injury.

**Results:**

SC improved survival from 0% to approximately 90% through day 14. Plasma IL-6 and C-reactive protein levels declined after the acute phase, and little to no bacterial burden was detected in peritoneal lavage fluid, blood, or lung homogenates on day 14. Despite apparent systemic recovery, SC mice exhibited persistent pulmonary inflammation, with elevated levels of lung inflammatory mediators and sustained accumulation of neutrophils and monocytes. In contrast, CD4^+^ T cells, CD8^+^ T cells, and B cells were reduced in the lungs on day 14. IgM and IgG were elevated in plasma and lung homogenates but remained unchanged in BALF. Conversely, IgA levels were preserved in plasma and lung homogenates but selectively reduced in BALF. SC mice also showed reduced airway epithelial expression of E-cadherin, Occludin, and polymeric immunoglobulin receptor (pIgR), suggesting epithelial injury and impaired airway IgA transport in the post-acute phase.

**Conclusions:**

We developed a clinically aligned preclinical model of source-controlled abdominal sepsis that enables evaluation of post-acute pathophysiology. Despite apparent systemic recovery, sepsis survivor animals exhibited persistent pulmonary inflammation, adaptive immune cell reduction, airway epithelial injury, reduced pIgR expression, and selective BALF IgA loss. These findings suggest prolonged disruption of airway mucosal immunity after abdominal sepsis.

**Supplementary Information:**

The online version contains supplementary material available at 10.1186/s40635-026-00931-3.

## Background

Advances in intensive care and evidence-based guidelines have improved the short-term survival of patients with sepsis, particularly in high-income settings [1]. Consequently, research focus has shifted toward long-term outcomes among survivors. Nevertheless, while approximately 50% of patients return to the workforce within two years of discharge [2], approximately 40% are readmitted within 90 days [3]. Among the various etiologies, abdominal sepsis is particularly severe, with a high incidence of chronic critical illness [4]; approximately 40% of patients with sepsis in the surgical intensive care unit eventually die within one year [5].

Many complications arise after sepsis, including immunoparalysis and increased susceptibility to infection, which contribute significantly to late mortality [6, 7]. The respiratory tract is the most common site of secondary infection [8–10], and pneumonia is the leading cause of readmission after sepsis [11–13]. These observations suggest that the lungs remain particularly vulnerable even beyond the acute phase. Understanding pulmonary immune alterations following sepsis could inform strategies for improving long-term outcomes.

To investigate these processes, preclinical models that allow longitudinal evaluation after abdominal sepsis are required. Cecal ligation and puncture (CLP) is widely used to develop a standard rodent model of polymicrobial sepsis because it recapitulates several key features of human sepsis, including systemic inflammation and multiorgan failure [14]. However, conventional CLP has important limitations for modeling the post-acute phase, particularly because the necrotic cecum remains in place and source control is not performed. This limits its alignment with the clinical management of abdominal sepsis and complicates the interpretation of long-term outcomes.

The Minimum Quality Thresholds in Pre-Clinical Sepsis Studies (MQTiPSS) consensus emphasizes that preclinical sepsis models should be designed according to the clinical condition being modeled and should incorporate clinically relevant features, such as appropriate infection sources, organ dysfunction endpoints, supportive care, antimicrobial therapy, and transparent reporting of limitations [15]. In line with these principles, several modified CLP models have incorporated source control and antibiotic treatment to better approximate clinical practice [16–18]. In addition, the use of older animals has been encouraged to improve relevance to human sepsis populations. Nevertheless, few studies have examined post-acute pulmonary immune alterations following source-controlled abdominal sepsis under clinically aligned conditions.

Here, we aimed to develop a modified CLP model incorporating delayed resection of the necrotic cecum, peritoneal lavage, and antibiotic therapy, allowing longitudinal assessment of sepsis survivors for up to two weeks. We used 28-week-old mice to avoid exclusive reliance on very young adult animals while recognizing that this age does not fully recapitulate the pathology of middle-aged or elderly human populations. Using this model, we aimed to characterize post-acute pulmonary inflammation and airway mucosal immune alterations after source-controlled abdominal sepsis.

## Methods

### Animals

Male C57BL/6 mice (28–30 weeks old; CLEA Japan, Tokyo, Japan) were maintained under specific pathogen-free conditions (25 °C, 40% humidity, 12-h light/dark cycle) with *ad libitum* access to food and water. All animal procedures were approved by the Ethics Committee of Animal Care and Experimentation of National Defense Medical College, Japan (approval number: 21062).

### Model establishment

**Fig. 1:**
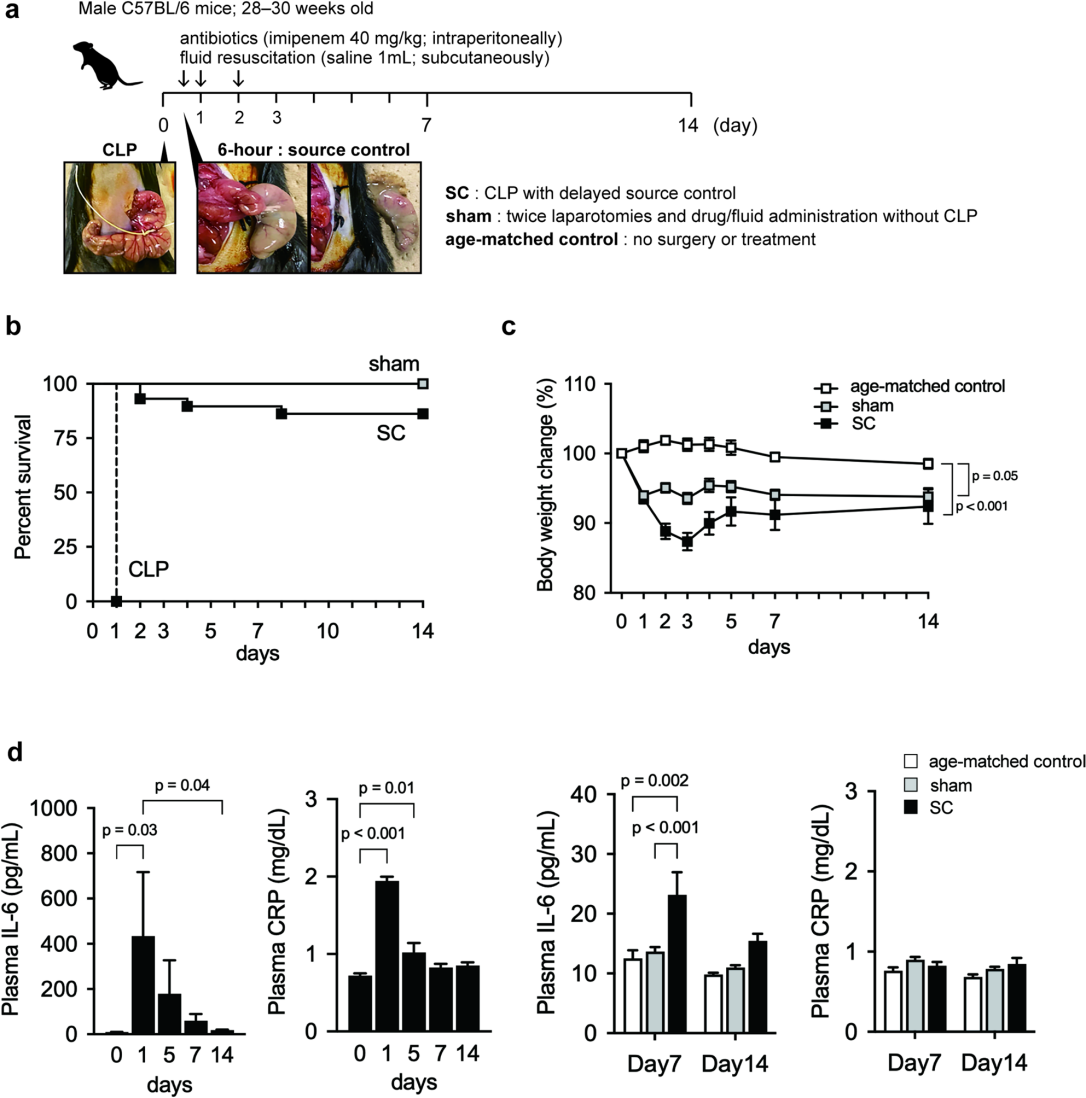
Murine model of CLP-induced abdominal sepsis with source control. **a**, Experimental design. **b**, Survival over 14 days for CLP, sham, and SC groups (CLP *n* = 5; sham *n* = 16; SC *n* = 30). **c**, Body–weight trajectories in age-matched control, sham, and SC mice. (*n* = 10 each). **d**, Plasma IL-6 and CRP levels in SC mice (*n* = 3–7/time point). Plasma IL-6 and CRP in each group on days 7 and 14 (*n* = 4–8/group). Data are represented as the mean ± SEM. CLP, cecal ligation and puncture; SC, source control; IL-6, interleukin-6; CRP, C-reactive protein

Mice were assigned to three groups: (i) CLP with delayed source control (SC), (ii) sham, and (iii) age-matched controls (Fig. 1a). Mice allocated to the SC and sham groups were randomly assigned before surgery using a random number-based allocation method.

SC group: Sepsis was induced by CLP. Under isoflurane anesthesia, a 1-cm incision was made in the left upper abdomen, and the cecum was ligated just below the ileocecal valve using a 1 − 0 silk suture and punctured once near the ligation site using an 18-gauge needle. After confirming leakage of the cecal contents, the cecum was replaced, and the abdomen was closed. In CLP models, body temperature < 30 °C or a > 5 °C drop from baseline is an endpoint [19]. As body temperature fell to 29.8 ± 2.1 °C by 6 h, source control was performed at this time point. The mice were re-anesthetized, and a 3-cm midline laparotomy was performed to remove the necrotic cecum distal to the ligation. The cavity was lavaged with 50 mL of warm saline and closed in layers. Before skin closure, imipenem (4 mg/kg; Merck, West Point, PA, USA), a broad-spectrum antibiotic, was administered intraperitoneally, and 1 mL of saline was administered subcutaneously for fluid resuscitation. The same doses of intraperitoneal imipenem and subcutaneous saline were administered on postoperative days 1 and 2.

Sham Group: Under identical conditions pertaining to anesthesia, timing, incisions, and drug/fluid administration, the cecum was exteriorized and replaced without ligation or puncture. At 6 h, a midline incision was made and closed immediately, followed by the same regimen of imipenem and saline.

Age-matched control group: Body weight and survival of age-matched mice, not subjected to surgery or treatment, were monitored for 14 days.

### Blood sampling and laboratory analysis

The mice were euthanized, and blood was collected from the inferior vena cava. Samples were centrifuged at 950 × *g* for 10 min at 4 °C, and plasma was stored at − 80 °C until analysis. Aspartate aminotransferase (AST), alanine aminotransferase (ALT), and blood urea nitrogen (BUN) levels were measured using a dry chemistry analyzer (FUJI DRI-CHEM3500V, FUJIFILM Corporation, Tokyo, Japan).

### Bacterial load analysis

On day 14, bacterial burden was assessed in peritoneal lavage fluid, blood, and lung homogenate from SC mice. Mice were euthanized, and the abdominal surface was disinfected. The peritoneal cavity was opened aseptically, 1 mL of sterile saline was injected intraperitoneally, and peritoneal lavage fluid was collected. Blood was collected from the inferior vena cava, and the lungs were harvested. Lung tissue was homogenized in sterile saline. Each sample was placed on brain heart infusion agar plates (EIKEN CHEMICAL CO., LTD., Tokyo, Japan) and incubated overnight at 37 °C. Bacterial colonies were counted and expressed as colony-forming units (CFU) per mL of peritoneal lavage fluid or blood, or as CFU per gram of lung tissue.

### Bronchoalveolar lavage fluid (BALF) and lung homogenate preparation

Mice were euthanized, the trachea was cannulated, and bronchoalveolar lavage was performed three times with 1 mL of phosphate-buffered saline. BALF was centrifuged at 500 × *g* for 5 min at 4 °C, and supernatants were stored at − 80 °C. After BALF collection, residual intravascular blood was removed by cardiac perfusion with 10 mL of saline, and the lungs were harvested. The left lung was homogenized in 1 mL of phosphate-buffered saline. The homogenate was centrifuged at 500 × *g* for 5 min at 4 °C, and the supernatant was stored at − 80 °C. The protein concentration was measured using a bicinchoninic acid protein assay kit (Thermo Fisher Scientific, Waltham, MA, USA).

### Cytokines and immunoglobulin measurements

Cytokines interleukin (IL)-6, monocyte chemoattractant protein-1 (MCP-1) (BD Biosciences, San Jose, CA, USA), and C-reactive protein (CRP), macrophage inflammatory protein-2 (MIP-2) (R&D Systems, Minneapolis, MN, USA) were quantified by ELISA. Immunoglobulins (IgM, IgG, and IgA) were measured using ELISA kits (Bethyl Laboratories, Montgomery, TX, USA).

### Histopathology and immunostaining

On day 14, the lungs, liver, and kidneys were fixed in 10% neutral buffered formalin, sectioned at 3 μm, and stained with hematoxylin and eosin (H&E). For frozen sections, the lungs were fixed in 4% paraformaldehyde for 2 days and embedded in OCT compound (Sakura Finetek, Tokyo, Japan). Samples were snap-frozen in liquid nitrogen-cooled 2-methylbutane and sectioned at 10 μm using a cryostat (Leica, Wetzlar, Germany). The sections were then incubated with primary antibodies (Table S1) for 1 h. Secondary detection was performed using an ImmPRESS HRP Reagent KIT (VECTOR LABORATORIES, Newark, CA, USA) or Alexa Fluor 594-conjugated goat anti-rat IgG. Nuclei were counterstained with hematoxylin or DAPI (4′,6-diamidino-2-phenylindole). All tissue section images were acquired using either an APX100-SU fluorescence microscope (EVIDENT, Tokyo, Japan) or a BZ-X710 microscope (Keyence, Osaka, Japan). For each slide, five randomly selected high-power fields (20× magnification) were captured and used for quantitative analysis. The number of B220- or myeloperoxidase (MPO)-positive cells was counted for each mouse. E-cadherin, Occludin, and Polymeric immunoglobulin receptor (pIgR) expression was quantified using ImageJ software (version 2.14.0/1.54 g). The percentage of these positive areas was calculated by measuring and normalizing the total epithelial area in each image. Quantitative histological and immunohistochemical analyses were performed by an investigator blinded to the group allocation. For each mouse, randomly selected fields were analyzed, and the mean value per mouse was used for statistical analysis.

### Flow cytometry

After blood collection, residual intravascular blood was removed, and the lungs were harvested. The lungs were blotted dry, finely minced with a razor blade, and passed through a stainless-steel mesh (0.05 × 200; pore size 0.077 mm; Amichu Metal Mesh, Osaka, Japan) in RPMI 1640 medium (Thermo Fisher Scientific) with 0.5% bovine serum albumin. The cell suspension was passed through a 40 μm cell strainer (Corning, Corning, NY) and centrifuged at 500 × *g* for 5 min at 4 °C. The pellet was resuspended, red blood cells were lysed with erythrocyte lysis buffer (Merck), passed through a 15-µm strainer (pluriSelect Life Science, Leipzig, Germany), and washed twice with RPMI 1640 containing 0.5% bovine serum albumin. The cells were incubated with an anti-CD16/32 antibody for Fc receptor blocking for 15 min, and then stained with fluorochrome–conjugated antibodies (Table S2) for 15–20 min. The flow cytometric gating strategies are shown in Fig. S2a and S2c.

For assessment of phagocytic/bactericidal activity, cells were incubated with pHrodo Red *S. aureus* BioParticles (Invitrogen, Waltham, MA) for 70 min at 37 °C and 5% CO_2_; controls were incubated for 70 min at 4 °C.

Flow cytometry was performed on a BD FACS Canto II (BD Bioscience), and data were analyzed using FlowJo 10.4 (BD Biosciences).

### Statistical analysis

Data are expressed as mean ± standard error of the mean (SEM). Sample sizes varied among experiments and are indicated in the figure legends. Normality and homogeneity of variance were assessed using the Shapiro–Wilk test and Brown–Forsythe test, respectively, when applicable. For comparisons among three or more groups, one-way ANOVA with Tukey’s multiple comparisons test was used when parametric assumptions were met. When these assumptions were not met or could not be reliably evaluated because of a small sample size, the Kruskal–Wallis test followed by Dunn’s multiple comparisons test was used. Survival analysis was conducted using the Kaplan–Meier method and compared using the log-rank test with Bonferroni correction. All analyses were performed using GraphPad Prism 9 (GraphPad Software, La Jolla, CA, USA); *p* < 0.05 was considered statistically significant.

## Results

### Source control performed 6 h after CLP improved survival and enabled long-term observation

Without source control, all CLP mice died within 24 h. Source control performed 12 h after CLP did not improve outcomes (data not shown), whereas intervention at 6 h resulted in approximately 90% survival for 14 days (Fig. 1b). Body weight in the SC group began to recover after day 3 but remained approximately 90% of baseline throughout the 14-day observation period and was lower than that of age-matched controls (Fig. 1c). Sham mice showed no mortality but also did not fully regain their baseline weight.

### SC mice showed resolution of acute systemic inflammation without overt hepatic or renal injury

In SC mice that underwent source control 6 h after CLP, plasma IL-6 and CRP levels peaked on day 1 and declined by day 5 (Fig. 1d). IL-6 levels remained higher in SC mice than in sham or age-matched control mice on day 7 but returned to comparable levels by day 14 (Fig. 1d). In contrast, no significant group differences were observed in CRP levels at the later time points examined (Fig. 1d). Little to no bacterial burden was detected in peritoneal lavage fluid, blood, or lung homogenates in SC mice on day 14 (Fig. S1a).

Because sepsis commonly causes hepatic and renal injury [20–22], we assessed plasma AST, ALT, and BUN levels. These values remained within normal ranges on days 7 and 14 in the SC group (Fig. S1b). Consistently, H&E staining on day 14 revealed no remarkable pathological changes in the liver or kidneys (Fig. S1c).

### Pulmonary inflammation persisted in SC mice through day 14

**Fig. 2:**
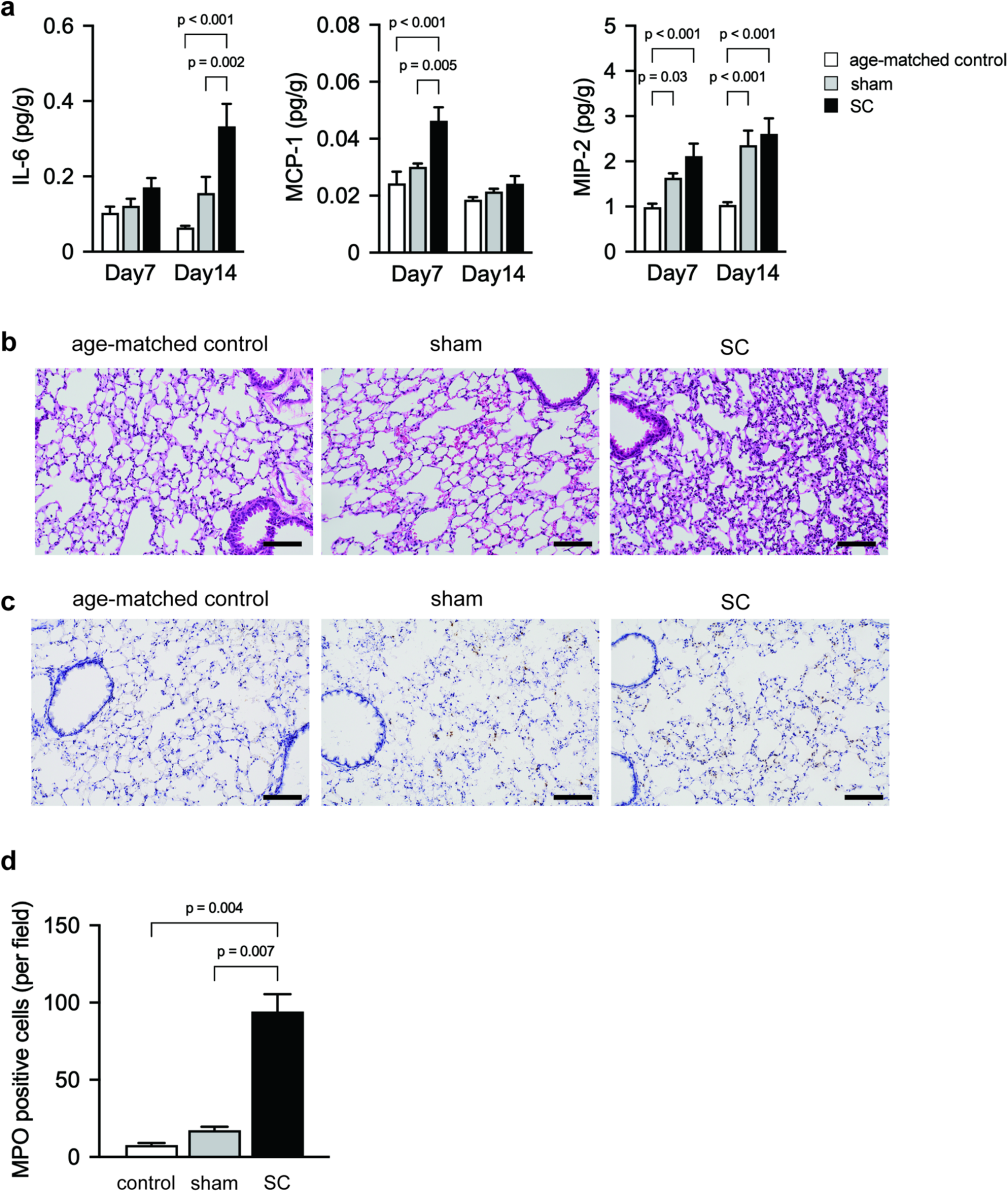
Pulmonary cytokines and histopathology. **a**, Lung homogenate concentrations of IL-6, MCP-1, and MIP-2 in age-matched control, sham, and SC mice on days 7 and 14. (*n* = 6–10/group). Representative lung sections on day 14: **b**, H&E staining showing inflammatory cell infiltration (arrows); **c**, MPO immunostaining showing MPO^+^ cells (arrows); scale bars, 100 μm. **d**, Quantification of MPO^+^ cells/field. Data are represented as the mean ± SEM. H&E, hematoxylin and eosin; MCP-1, monocyte chemoattractant protein-1; MIP-2, macrophage inflammatory protein-2; MPO, myeloperoxidase

In lung homogenates, IL-6 levels were the highest in SC mice on day 14. MCP-1 levels increased on day 7 in SC mice and returned to baseline by day 14. MIP-2 levels were elevated in SC mice on days 7 and 14 compared to those in age-matched controls, and sham mice also showed higher MIP-2 levels than age-matched controls at both time points (Fig. 2a). Histological analysis showed marked cell infiltration (Fig. 2b) and MPO-positive cells in SC lungs (Fig. 2c, d).

### SC mice exhibited sustained myeloid cell accumulation in the lungs

**Fig. 3:**
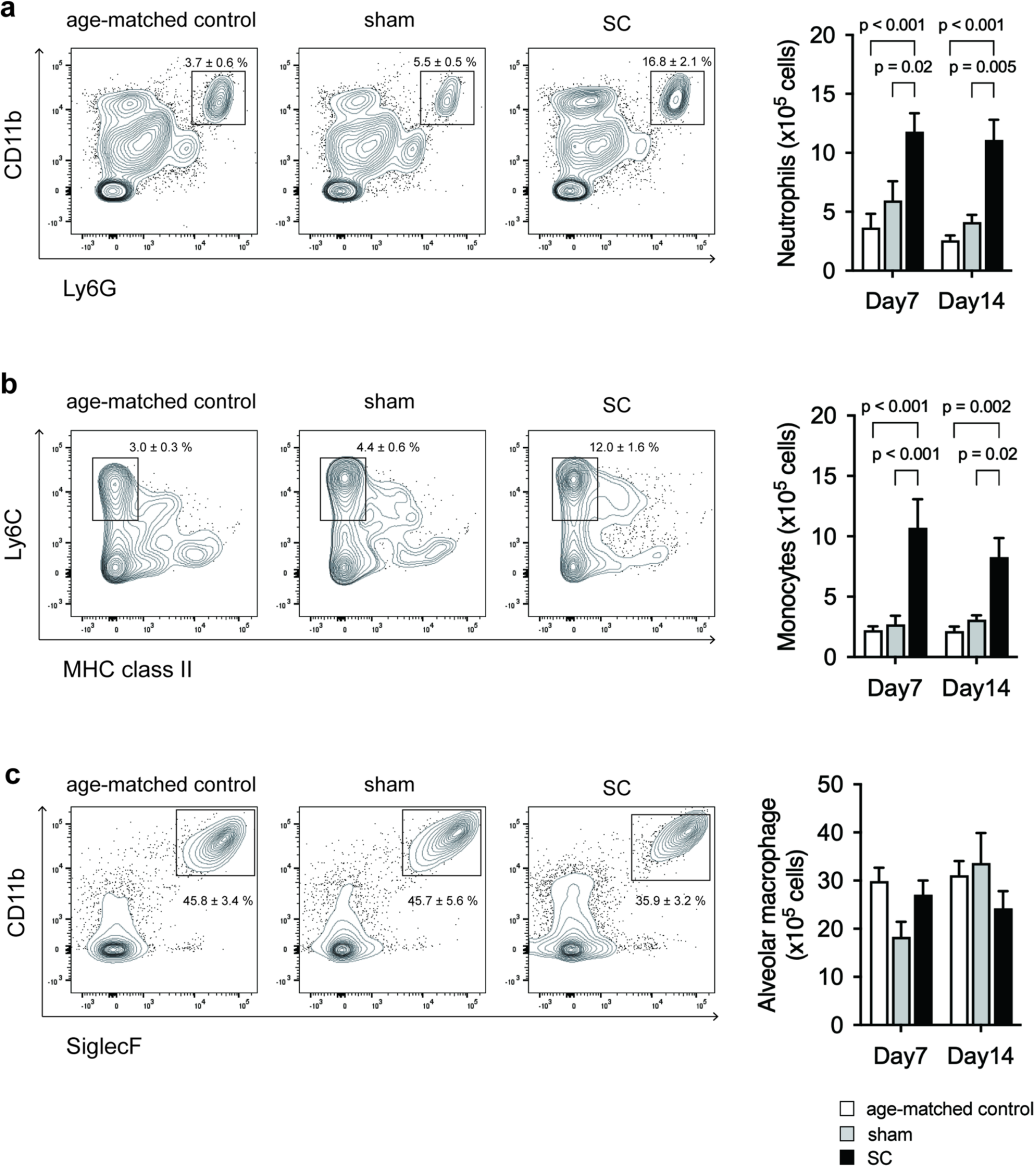
Flow cytometric assessment of pulmonary myeloid cells. Representative contour plots (percent among CD45^+^) and quantification of (**a**) neutrophils, (**b**) monocytes, and (**c**) alveolar macrophages in the lungs from control, sham, and SC mice on days 7 and 14 (*n* = 5–9/group). Data are represented as the mean ± SEM

Flow cytometry revealed higher neutrophil and monocyte counts in the lungs of SC mice on days 7 and 14 than in the lungs of sham and age-matched controls (Fig. 3a, b). The number of alveolar macrophages did not differ among the SC, sham, and age-matched control groups (Fig. 3c). Phagocytic/bactericidal activity on day 14 did not differ between SC and age-matched controls for either neutrophils or alveolar macrophages (Fig. S2b). These findings suggest that prolonged pulmonary inflammation in SC mice reflects the sustained accumulation of neutrophils and monocytes rather than an overt enhancement of cell phagocytic function.

### SC mice had fewer lung T and B cells at day 14

**Fig. 4:**
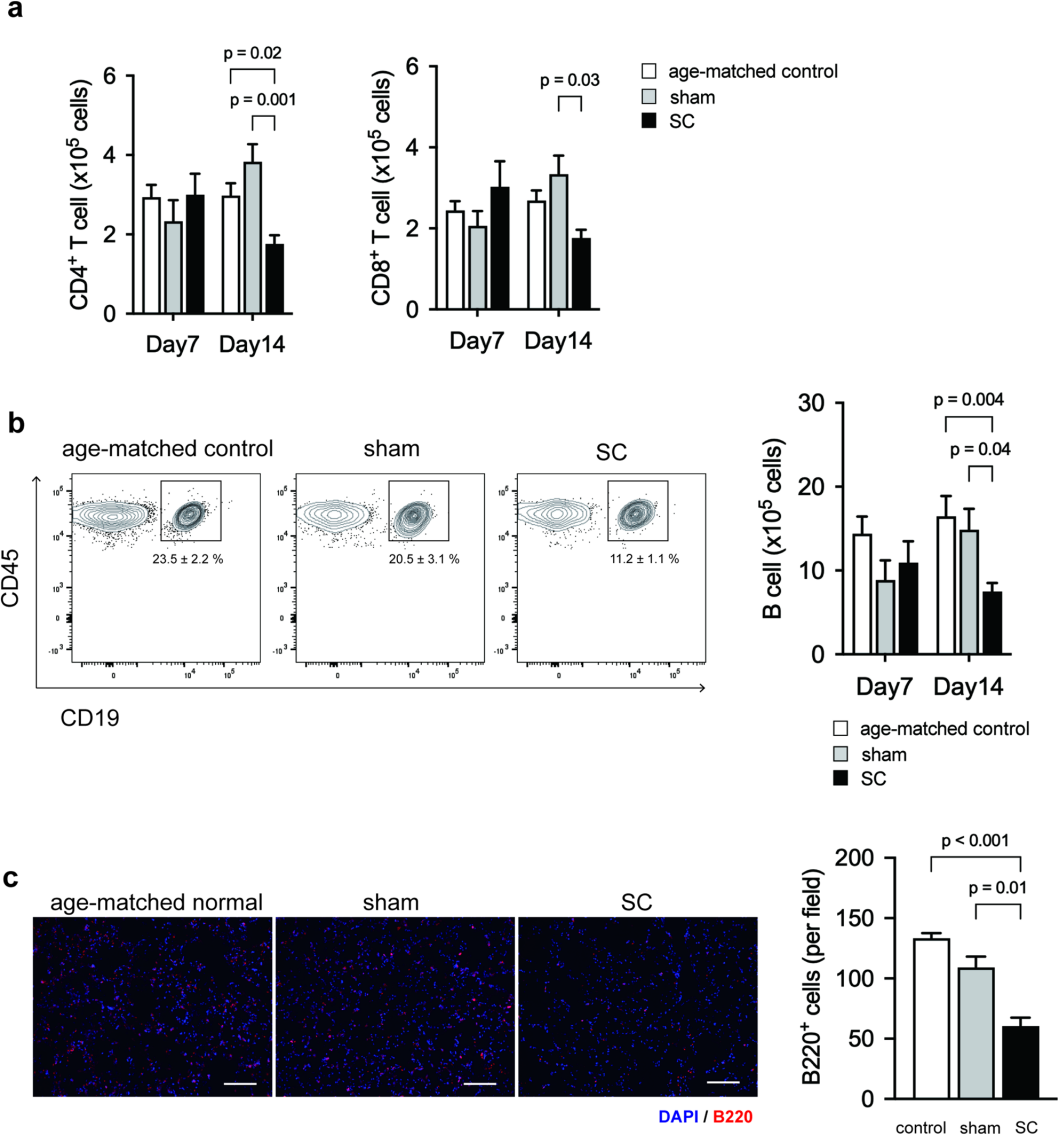
Flow cytometric and histological assessment of pulmonary lymphoid cells. Representative plots (percent among CD45^+^) and quantification of (**a)** CD4^+^/CD8^+^ T cells and (**b**) B cells in the lungs of control, sham, and SC mice on days 7 and 14 (*n* = 5–9/group). **c**, B220 immunostaining of lungs on day 14; scale bars, 100 μm. Quantification of B220^+^cells/field. Data are represented as the mean ± SEM

Next, we analyzed the distribution of adaptive immune cells in lung tissue. In SC mice, the numbers of CD4^+^ and CD8^+^ T cells were significantly lower than those in sham mice on day 14 but not on day 7 (Fig. 4a). The number of B cells was also lower than that in the age-matched controls (Fig. 4b). Sham mice did not differ significantly from the age-matched controls. Immunostaining confirmed B-cell loss in the lungs of SC mice (Fig. 4c). The pulmonary and splenic plasma cell frequencies remained unchanged among the groups (Fig. S2c).

### SC mice showed higher IgM and IgG levels in plasma and lung tissue but lower BALF IgA levels on day 14

**Fig. 5:**
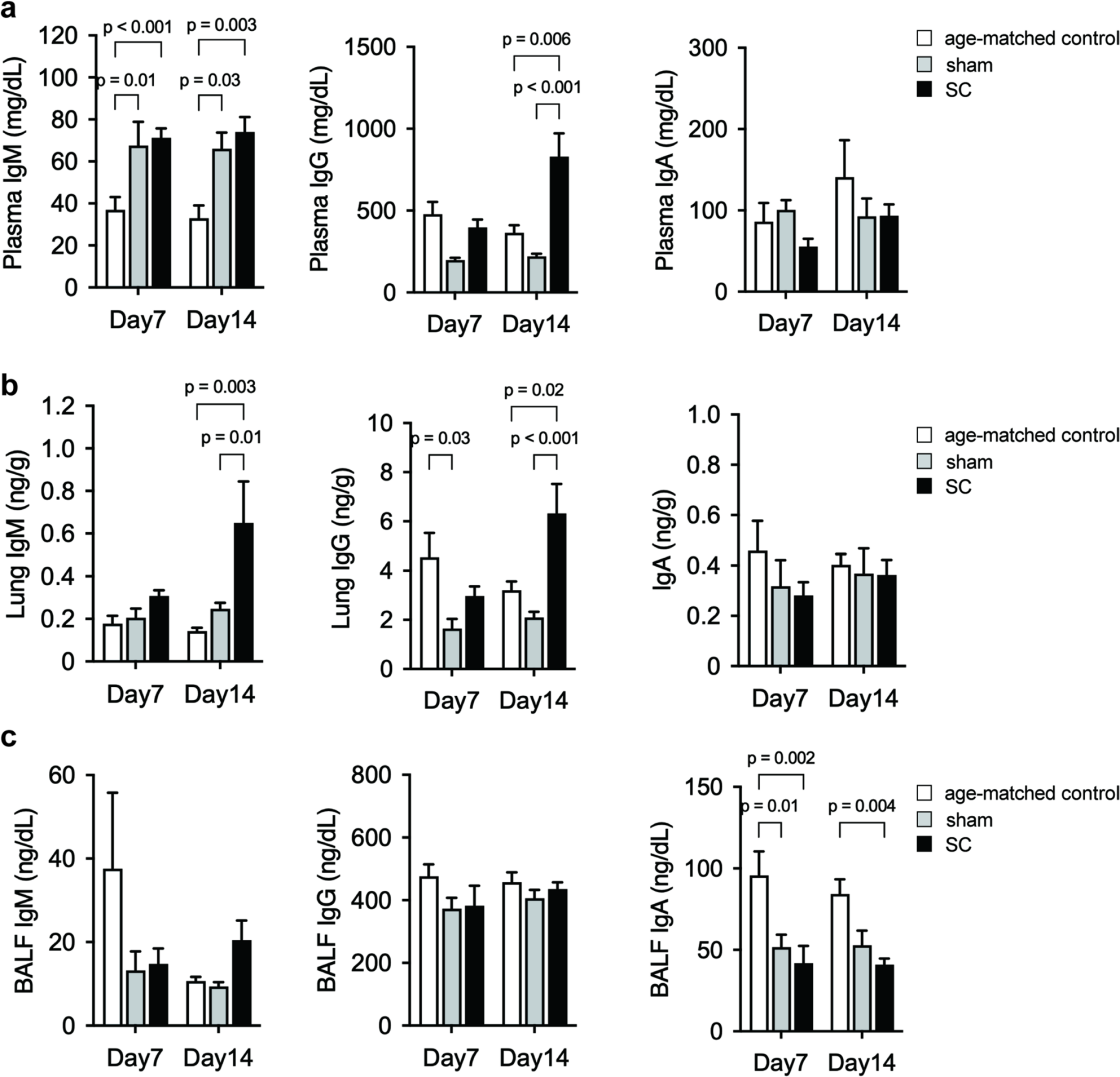
Immunoglobulins in plasma, lung, and BALF. **a**, Plasma immunoglobulins (IgM, IgG, and IgA) on days 7 and 14. **b**, Immunoglobulins in left-lung homogenates. **c**, Immunoglobulins in BALF from control, sham, and SC mice (*n* = 4–10/group). Data are represented as the mean ± SEM. BALF, bronchoalveolar lavage fluid

**Fig. 6:**
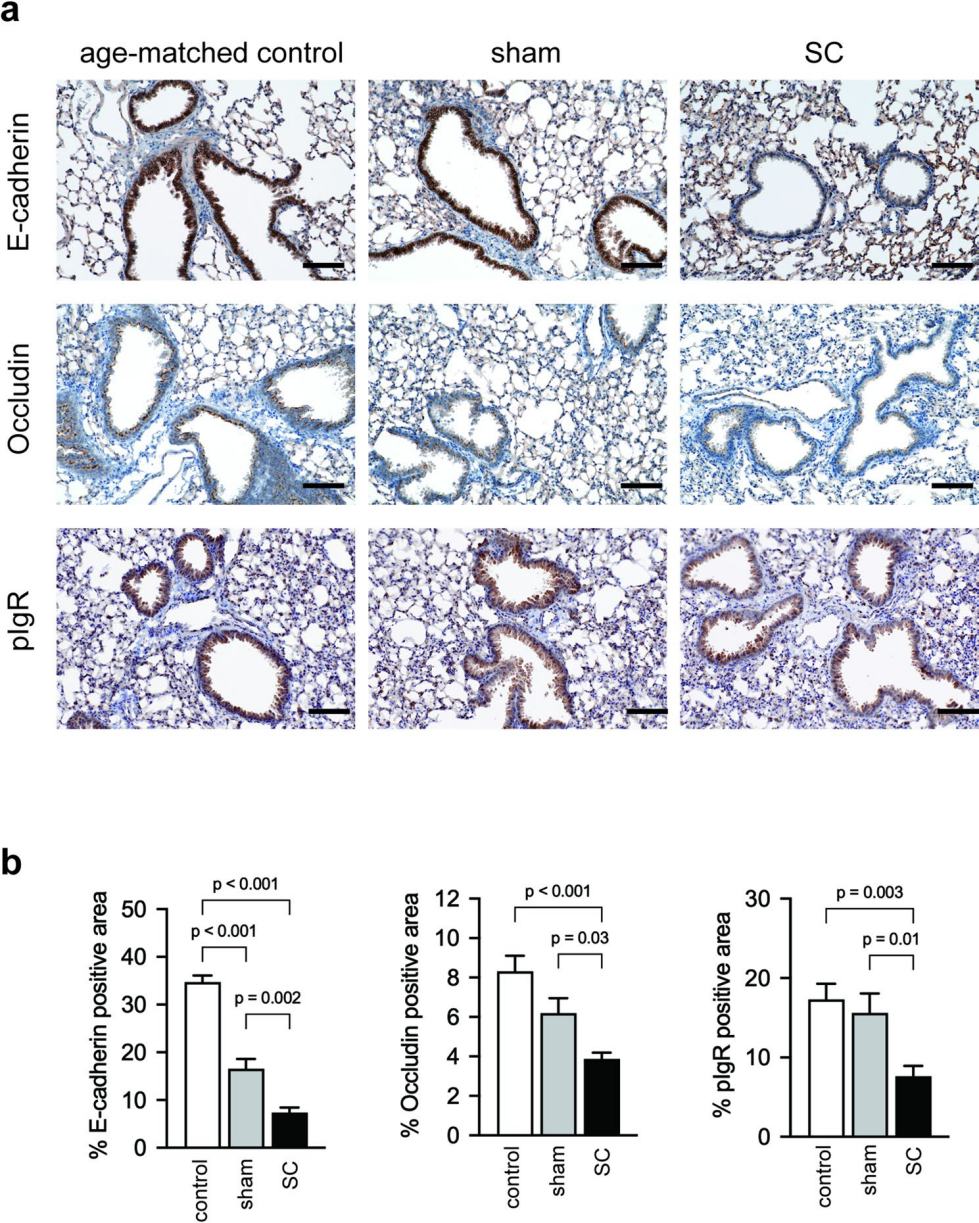
Immunostaining images in airway (tracheal) epithelium on day 14 and quantitation of positive area; **a**, Representative images of E-cadherin, Occludin, and pIgR staining in the airway (tracheal) epithelium (×20).; scale bars, 100 μm. **b**, Quantification of the positive area for E-cadherin, Occludin, and pIgR. Data are represented as the mean ± SEM. pIgR, polymeric immunoglobulin receptor

SC mice exhibited higher plasma IgM (days 7 and 14) and IgG (day 14) levels than age-matched controls, whereas IgA levels were unchanged. Sham mice also showed higher plasma IgM levels than age-matched controls, whereas plasma IgG levels did not differ between the two groups (Fig. 5a). In lung homogenates, SC mice had higher IgM and IgG levels on day 14, whereas IgA levels remained unchanged (Fig. 5b). In BALF, IgM and IgG levels were unchanged among groups, but IgA levels were significantly reduced in SC mice (days 7 and 14) compared to age-matched controls. Sham mice also exhibited lower BALF IgA levels than age-matched controls on day 7 (Fig. 5c). On day 14, the expression of E-cadherin, Occludin, and pIgR in the tracheal epithelium was lower in SC mice than in age-matched controls. Sham mice also showed lower E-cadherin expression than age-matched controls, whereas Occludin and pIgR expression did not significantly differ between sham mice and the age-matched controls (Fig. 6a, b).

## Discussion

In this study, we established a clinically aligned murine CLP model incorporating source control and antibiotic treatment, enabling analysis of post-sepsis pathophysiology beyond the acute phase. Source control markedly improved survival, reduced systemic inflammation, and was associated with little to no detectable bacterial burden in peritoneal lavage fluid, blood, or lung homogenates by day 14. Nevertheless, pulmonary inflammation persisted and was characterized by elevated inflammatory mediators, sustained myeloid cell accumulation, epithelial injury, and selective reduction of BALF IgA. Importantly, this model is intended to capture selected post-acute pulmonary immune alterations after source-controlled abdominal sepsis, rather than to fully recapitulate the complex clinical phenotype of human intensive care unit sepsis. Within this scope, our findings suggest that source-controlled abdominal sepsis can induce prolonged pulmonary immune alterations even after apparent systemic recovery, which may be relevant to the vulnerability of sepsis survivors to secondary respiratory infections.

Infection control is crucial for the management of clinical sepsis. Patients with severe abdominal sepsis usually require source control of infection foci, peritoneal lavage, and antimicrobial therapy [23]. Consistent with the MQTiPSS framework, our model incorporated these clinically relevant elements to improve alignment with the management of human abdominal sepsis while recognizing that no animal model can fully recapitulate the complexity of sepsis observed in patients in the intensive care units. In this study, delayed cecal resection 6 h after CLP, combined with peritoneal lavage and antibiotic treatment, markedly improved survival in an otherwise lethal model. This finding is consistent with previous reports showing that surgical removal of the necrotic cecum after CLP improves survival and attenuates systemic inflammation during the acute phase of CLP-induced sepsis [24, 25]. We further demonstrated that plasma IL-6 and CRP levels in SC mice declined after the acute phase and returned to levels comparable to controls by day 14. In addition, little to no bacterial burden was detected in peritoneal lavage fluid, blood, or lung homogenates on day 14. Although the initial CLP insult was comparable in severity to previous severe CLP models [26, 27], bacterial burden was relatively very low or undetectable in SC mice. These results suggest that source control combined with antibiotic treatment improved survival, contributed to effective microbial control, and alleviated sustained systemic inflammation in septic mice in the post-acute phase.

Despite this apparent systemic recovery, pulmonary inflammation persisted. Sepsis induces pulmonary inflammatory responses in mouse models; however, most previous studies have focused on the acute phase within 36 h after sepsis induction [28, 29]. In contrast, the present model showed that inflammatory mediators, including IL-6, MCP-1, and MIP-2, remained elevated in the lung tissue during the post-acute phase, accompanied by sustained accumulation of neutrophils and monocytes. However, some inflammatory changes, particularly elevated MIP-2 level, were also observed in sham mice, indicating that repeated laparotomy and surgical stress may have contributed to part of the pulmonary inflammatory phenotype. Notably, bacterial burden was minimal or undetectable in lung homogenates on day 14, suggesting that persistent pulmonary inflammation cannot be simply explained by overt ongoing bacterial infection in the lung. These findings may be relevant to clinical observations that sepsis survivors are at high risk of pulmonary complications and pneumonia-related readmission after hospital discharge [11–13]. However, the mechanisms underlying the induction of persistent pulmonary inflammation via abdominal sepsis after source control remain to be elucidated.

A hallmark of sepsis-induced immunoparalysis [30] is lymphocyte dysfunction [31, 32], and sepsis promotes apoptosis and depletion of both T and B cells [33–35]. In the present model, CD4⁺ T cells, CD8⁺ T cells, and B cells were reduced in the lung on day 14, consistent with previous acute-phase studies showing sepsis-induced lymphocyte loss [36, 37]. These findings suggest that adaptive immune alterations in the lung may persist beyond apparent systemic recovery. In contrast, IgM and IgG levels were elevated in plasma and lung homogenates, while the frequencies of plasma cells in the lung and spleen did not indicate a generalized loss of immunoglobulin-producing capacity. The selective reduction of IgA in BALF, despite preserved IgA levels in plasma and lung homogenates, suggests impaired mucosal transport, secretion, or compartmentalization rather than a global defect in IgA production.

This interpretation is consistent with the role of pIgR-dependent IgA transport in airway mucosal immunity. IgA plays an essential role in host defense by preventing pathogen adherence and maintaining immune homeostasis at mucosal surfaces [38, 39]. Because CLP induces polymicrobial peritonitis rather than infection with a defined bacterial pathogen, we did not assess antigen-specific immunoglobulin responses. Therefore, the functional specificity of the immunoglobulin changes observed in this study remains unclear. Nevertheless, the persistent reduction in BALF IgA may reflect weakened mucosal defense at the airway surface and could potentially increase susceptibility to inhaled pathogens. In this context, the reduced expression of pIgR in the airway epithelium of SC mice is particularly relevant. Because pIgR mediates epithelial transport of polymeric IgA into the airway lumen, reduced pIgR expression may be associated with the selective reduction of BALF IgA despite preserved IgA levels in plasma and lung homogenates. Moreover, reduced expression of E-cadherin and Occludin suggests persistent airway epithelial injury or barrier disruption, which may also be associated with altered airway IgA transport. Similar associations among airway epithelial injury, reduced pIgR expression, and impaired mucosal immunity have been reported in acute respiratory distress syndrome [40] and chronic obstructive pulmonary disease [41]. Thus, our data support an association among epithelial injury, reduced pIgR expression, and selective BALF IgA loss, but do not establish a direct causal pathway. Taken together, these findings suggest that source-controlled abdominal sepsis induces persistent pulmonary inflammation and epithelial injury, accompanied by impaired airway IgA transport. Such alterations may contribute to long-lasting disruption of airway mucosal immunity, although their functional consequences for susceptibility to secondary pneumonia remain to be determined.

The findings of this study should be interpreted in light of several limitations. First, sham mice underwent two laparotomies to match the surgical burden of the SC group. Because surgical stress alone can alter immune parameters, including circulating B cells and neutrophils [42], some changes observed in sham mice, such as reduced BALF IgA and elevated MIP-2, may reflect the biological effects of repeated surgery. Therefore, differences between SC and sham mice should be interpreted as sepsis-associated changes occurring in the context of surgical stress, rather than as effects of sepsis alone. In addition, induction of CLP itself required laparotomy, but clinical abdominal sepsis does not arise through an experimentally imposed initial surgical insult. Thus, operative stress is inherent to this model from the time of sepsis induction and should be considered when interpreting its translational relevance. Second, although this model incorporates clinically relevant elements, including source control, peritoneal lavage, and antibiotic treatment, it does not fully recapitulate the complexity of human ICU sepsis. Approximately 90% survival rate recorded in our SC model is higher than that observed in many severely ill clinical populations. This difference can be attributed to several factors, including the use of 28-week-old mice, which do not represent middle-aged or elderly human patients, and the relatively short 6-h delay before source control. Third, although bacterial burden was minimal or undetectable in the samples examined at day 14, we did not perform continuous longitudinal bacterial cultures at all time points or assess all possible tissue reservoirs. Finally, we did not perform a secondary pulmonary infection challenge; therefore, whether the observed pulmonary immune alterations directly increase susceptibility to pneumonia remains to be determined.

## Conclusion

We developed a source-controlled murine CLP model that permits post-acute evaluation of sepsis survivors. Although systemic inflammation and bacterial burden were largely resolved by day 14, pulmonary inflammation, epithelial injury, adaptive immune cell reduction, and selective BALF IgA loss persisted. These findings suggest that source-controlled abdominal sepsis can induce prolonged airway mucosal immune alterations, and the developed model provides a useful platform for investigating mechanisms underlying respiratory vulnerability after sepsis.

## Supplementary Information

Supplementary Material 1. Figure S1. Bacterial load, organ dysfunction markers, and histopathology. a, Bacterial load in peritoneal lavage fluid, blood, and lung homogenates from SC mice on day 14 (n=4). Data are represented as the median with interquartile range. b, Plasma AST, ALT, and BUN levels in the control, sham, and SC mice on days 7 and 14 (n = 6–10/group) Data are represented as the mean ± SEM. c, Representative H&E sections of liver and kidney on day 14; scale bars, 100 µm. SC, source control; AST, aspartate aminotransferase; ALT, alanine aminotransferase; BUN, blood urea nitrogen; H&E, hematoxylin and eosin.

Supplementary Material 2. Figure S2. Gating strategies and bactericidal activities. a, Gating strategy for lung-cell panel. b, pHrodo-based phagocytic/bactericidal activity of neutrophils and alveolar macrophages in age-matched control and SC mice on day 14. c, Frequencies of plasma cells among CD45+ cells in lung and spleen on day 14; plasma cells were defined as CD19⁻CD138⁺ cells. Data are represented as the mean ± SEM.

Supplementary Material 3. Table S1. List of antibodies used for immunohistochemistry. Table S2. List of antibodies used for flow cytometric analysis.

## Data Availability

The datasets generated and/or analyzed during the current study are available from the corresponding author on reasonable request.

## Abbreviations

ALT: Alanine aminotransferase
AST: Aspartate aminotransferase
BALF: Bronchoalveolar lavage fluid
BUN: Blood urea nitrogen
CLP: Cecal ligation and puncture
CRP: C-reactive protein
IL-6: Interleukin-6
MCP-1: Monocyte chemoattractant protein-1
MIP-2: Macrophage inflammatory protein-2
MPO: Myeloperoxidase
pIgR: Polymeric immunoglobulin receptor
SC: Source control
SEM: Standard error of the mean
