# Supplementary figures and images for: Source-controlled bacterial peritonitis improves survival but leaves persistent lung inflammation and airway IgA loss
Source: Intensive Care Med Exp. 2026 Jun 22;14:78. doi: 10.1186/s40635-026-00931-3 (PMC13287290; doi:10.1186/s40635-026-00931-3)

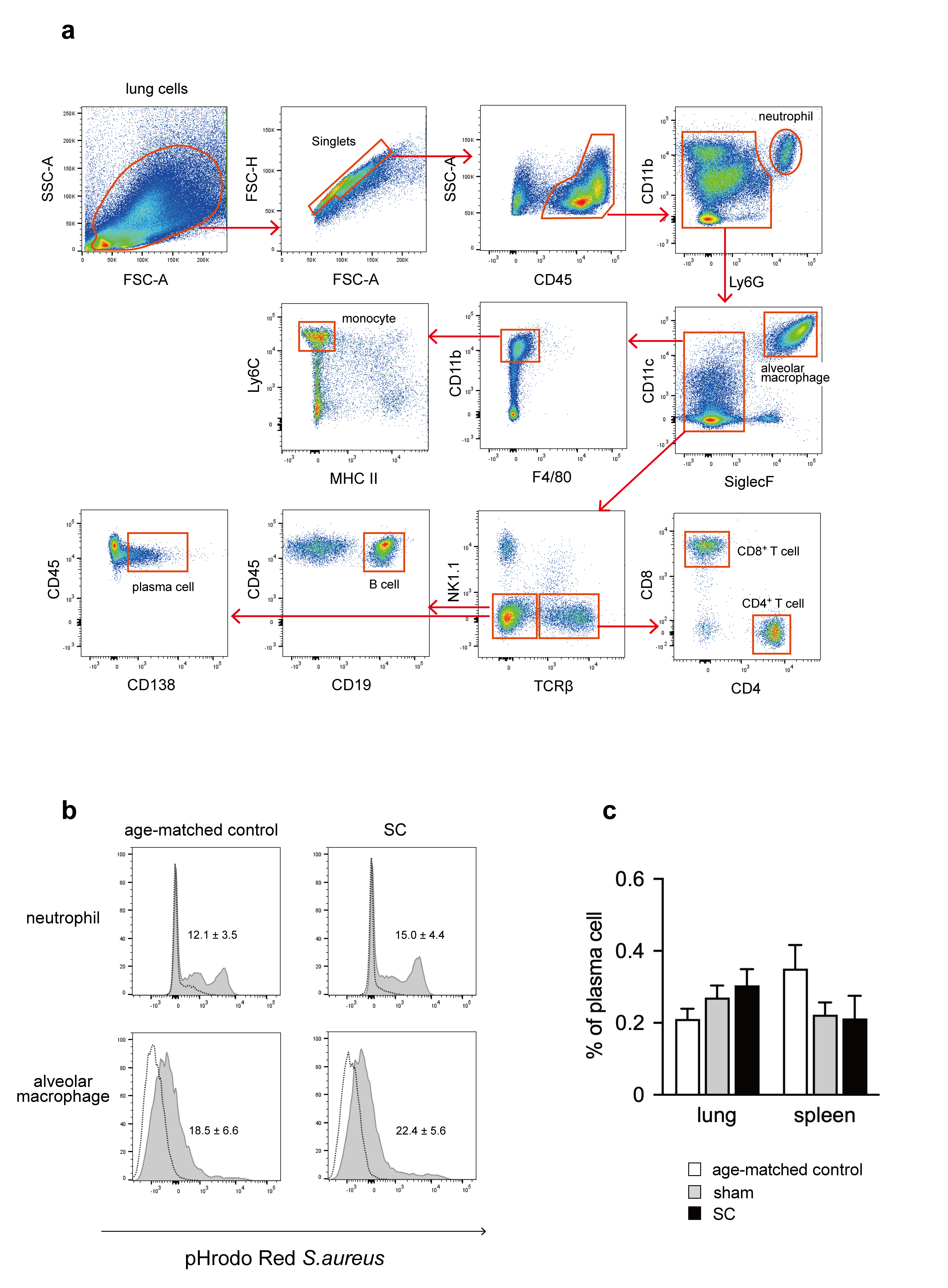

Supplement: Supplementary file 1 — Supplementary Material 1. Figure S1. Bacterial load, organ dysfunction markers, and histopathology. a, Bacterial load in peritoneal lavage fluid, blood, and lung homogenates from SC mice on day 14 (n=4). Data are represented as the median with interquartile range. b, Plasma AST, ALT, and BUN levels in the control, sham, and SC mice on days 7 and 14 (n = 6–10/group) Data are represented as the mean ± SEM. c, Representative H&E sections of liver and kidney on day 14; scale bars, 100 µm. SC, source control; AST, aspartate aminotransferase; ALT, alanine aminotransferase; BUN, blood urea nitrogen; H&E, hematoxylin and eosin. [file 40635_2026_931_MOESM1_ESM.tif]

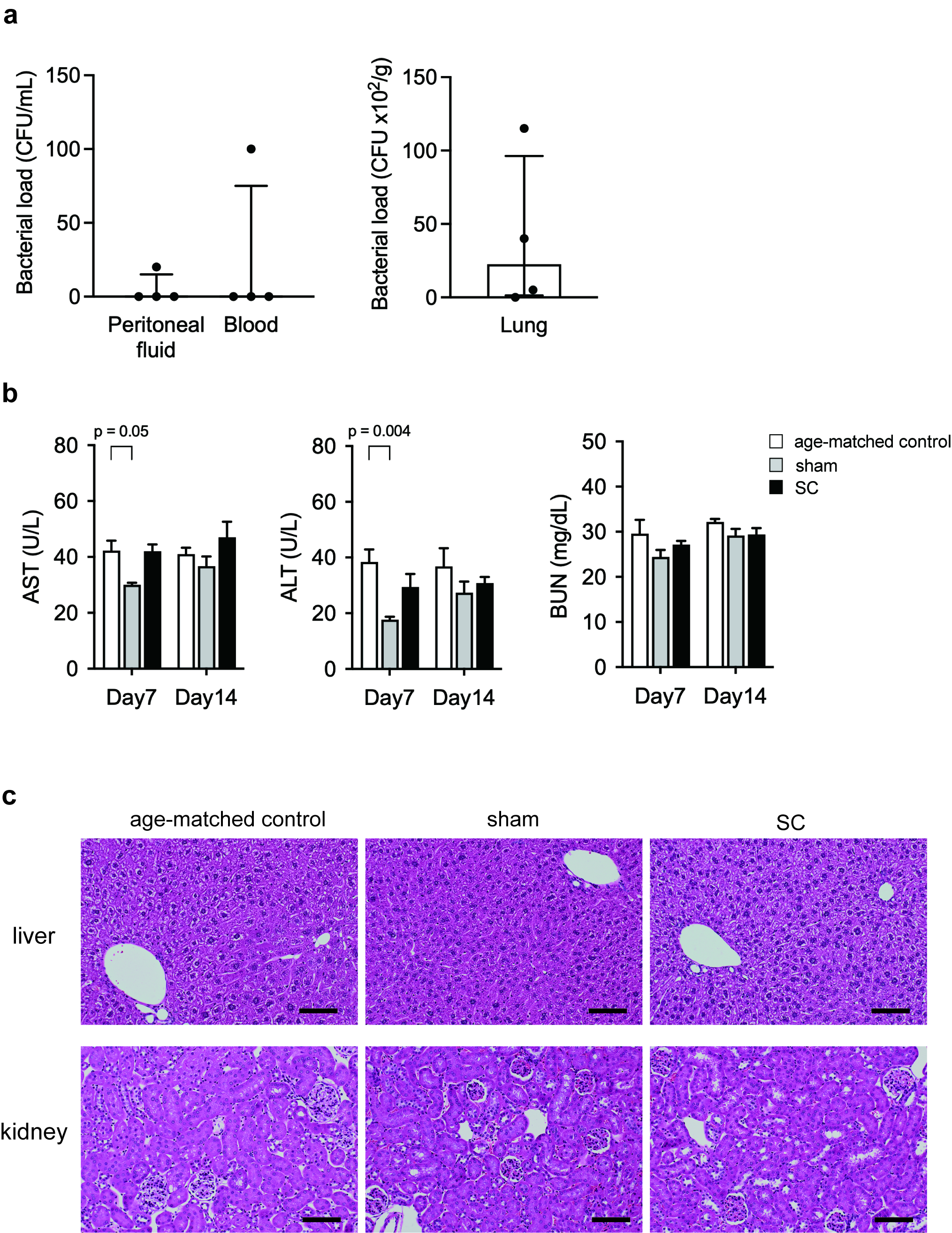

Supplement: Supplementary file 2 — Supplementary Material 2. Figure S2. Gating strategies and bactericidal activities. a, Gating strategy for lung-cell panel. b, pHrodo-based phagocytic/bactericidal activity of neutrophils and alveolar macrophages in age-matched control and SC mice on day 14. c, Frequencies of plasma cells among CD45+ cells in lung and spleen on day 14; plasma cells were defined as CD19⁻CD138⁺ cells. Data are represented as the mean ± SEM. [file 40635_2026_931_MOESM2_ESM.tif]
