# Supplementary material for: Source-controlled bacterial peritonitis improves survival but leaves persistent lung inflammation and airway IgA loss
Source: Intensive Care Med Exp. 2026 Jun 22;14:78. doi: 10.1186/s40635-026-00931-3 (PMC13287290; doi:10.1186/s40635-026-00931-3)
Supplement: Supplementary file 3 — Supplementary Material 3. Table S1. List of antibodies used for immunohistochemistry. Table S2. List of antibodies used for flow cytometric analysis. [file 40635_2026_931_MOESM3_ESM.doc]

Supplemental Table 1. List of antibodies and reagents used for immunohistochemistry

| Antibodies and Reagents | Source | Clone | Identifier | Dilution |
| --- | --- | --- | --- | --- |
| Rabbit anti-Myeloperoxidase | abcam | EPR20257 | ab208670 | 1:1000 |
| Mouse anti-E-Cadherin | Cell Signaling | 4A2 | 14472 | 1:2000 |
| Rabbit anti-Occludin | Abcam | EPR20992 | ab216327 | 1:1000 |
| Rabbit anti-Polymeric Immunoglobulin Receptor | proteintech |  | 22024-1-AP | 1:100 |
| Rat anti-CD45R | BD Pharmingen | RA3-6B2 | 557390 | 1:100 |
| ImmPRESS Reagent, Anti-Rabbit IgG | VECTOR LABORATORIES |  | MP-7401-15 |  |
| ImmPRESS Reagent, Anti-Mouse IgG | VECTOR LABORATORIES |  | MP-7452-15 |  |
| M.O.M Blocking Reagent | VECTOR LABORATORIES |  | MKKB-2213-1 |  |
| Goat anti-rat IgG (H+L) cross-absorbed secondary antibody, Alexa Fluor 594 | Invitrogen |  | A11007 | 1:100 |
| 4’,6-diamidino-2-phenylindole (DAPI) | Dojindo |  | 342-07431 | 1:1000 |

Supplemental table2. List of antibodies used for flowcytometric analysis

| Antibody | Source | Clone | Identifier | Dilution |
| --- | --- | --- | --- | --- |
| FITC anti-mouse MHC class II antibody | BioLegend | M5/114.15.2 | 107605 | 1:540 |
| Alexa Fluor 488 anti-mouse TCRβ antibody | BioLegend | H57-597 | 109216 | 1:360 |
| PE anti-mouse CD11c antibody | BioLegend | N418 | 117307 | 1:360 |
| PE anti-mouse CD138 antibody | BioLegend | 281-2 | 142501 | 1:180 |
| PE/Cy7 anti-mouse SiglecF antibody | Invitrogen | 1RNM44N | 25-1702-82 | 1:180 |
| PE/Cy7 anti-mouse CD19 antibody | BioLegend | 1D3/CD19 | 152417 | 1:180 |
| APC anti-mouse F4/80 antibody | Invitrogen | BM8 | MF48005 | 1:180 |
| APC anti-mouse TCRβ antibody | Invitrogen | H57-597 | 17-5961-82 | 1:300 |
| APC/Cy7 anti-mouse Ly6G antibody | BioLegend | 1A8 | 127624 | 1:360 |
| APC/Cy7 anti-mouse CD4 antibody | BioLegend | 53-6.7 | 100413 | 1:300 |
| PerCP/Cy5.5 anti-mouse CD11b antibody | Invitrogen | M1/70 | 45-0112-82 | 1:360 |
| PerCP/Cy5.5 anti-mouse CD8 antibody | BioLegend | GK1.5 | 100733 | 1:300 |
| BV421 anti-mouse CD45 antibody | BioLegend | 30-F11 | 103134 | 1:540 |
| BV421 anti-mouse NK1.1 antibody | BioLegend | PK136 | 108741 | 1:180 |
| BV510 anti-mouse Ly6C antibody | BioLegend | 30-F11 | 128033 | 1:180 |
| BV510 anti-mouse CD45 antibody | BioLegend | HK11.4 | 103138 | 1:240 |
| anti-mouse CD16/32 antibody | eBioscience | 93 | 14-0161-82 |  |
